# Interkingdom microbiome physiology in colorectal cancer: barrier dysfunction, immune-metabolic signaling, and therapeutic resistance

**DOI:** 10.3389/fphys.2026.1922297

**Published:** 2026-09-07

**Authors:** Shamimeh Pourbahrighesmat, Alireza Tojjari, George Laliotis, Anwaar Saeed

**Affiliations:** 1 Department of Medicine, Division of Hematology & Medical Oncology, University of Pittsburgh Medical Center (UPMC), Pittsburgh, PA, United States; 2 University of Pittsburgh Medical Center (UPMC) Hillman Cancer Center, Pittsburgh, PA, United States

**Keywords:** colorectal cancer, epithelial barrier, gut microbiome, immune metabolism, interkingdom interactions, mycobiome, phageome, therapeutic resistance

## Abstract

Colorectal cancer develops within a physiologically altered intestinal ecosystem. Epithelial barrier disruption, chronic inflammatory signaling, immune remodeling, and metabolic rewiring reshape host-microbial interactions in this niche. Although most colorectal cancer microbiome reviews have emphasized bacterial taxa, the gut ecosystem is inherently multi-kingdom, comprising bacteria, fungi, bacteriophages, and other viral elements that interact with one another and with the host. In this review, we propose that the clinically relevant unit of the colorectal cancer microbiome is often not a single organism but a configured interkingdom whose functional output depends on local physiology. Throughout, we distinguish human observational associations from mechanisms demonstrated in experimental models and from prospectively validated clinical effects. We synthesize evidence linking the bacteriome, mycobiome, and phageome to colorectal carcinogenesis, immune modulation, metabolic signaling, and therapeutic response. We highlight context-dependent mechanisms, including the paradoxical effects of *Fusobacterium nucleatum*, fungal regulation of myeloid and inflammasome pathways, phage-mediated control of bacterial virulence, and cross-kingdom metabolic circuits involving butyrate, succinate, bile acids, lactate, and arginine. We further propose, as a conceptual framework requiring longitudinal validation, that therapeutic resistance may sometimes reflect a treatment-selected ecological state rather than the action of a single resistance-conferring microbe. Finally, we address the reproducibility challenges raised by low-biomass intratumoral microbiome studies and outline minimum methodological standards for future multi-kingdom investigations. A framework centered in physiology, attentive to contamination, and organized around interactions may improve biomarker development and support rational microbiome-directed strategies in colorectal cancer.

## 1 Introduction

Colorectal cancer (CRC) ranks as the second leading cause of cancer-related mortality globally (Du et al., 2025). Although immune-checkpoint inhibitors have transformed the treatment of mismatch-repair-deficient or microsatellite-instability-high (dMMR/MSI-H) tumors, most patients with CRCs are microsatellite-stable (MSS) and remain refractory to immunotherapy (Andre et al., 2020; Wang X. et al., 2024). In parallel, the rising incidence of early-onset CRC cannot be explained by germline genetics alone, underscoring the need to examine environmental and host-microbial determinants of disease biology (Gazzaniga and Kasper, 2025; Luevano et al., 2025). These clinical challenges converge on a central physiological question: how does the intestinal ecosystem and microenvironment regulate colorectal carcinogenesis, immune responsiveness, and therapeutic resistance?

CRC does not develop within an isolated epithelial compartment, but within a tumor-associated intestinal niche shaped by barrier disruption and mucus remodeling, chronic inflammation, immune-cell redistribution, and metabolic changes including hypoxia, and metabolic rewiring (Zhang et al., 2023; Chen Y. et al., 2025; Dong et al., 2026). These changes influence which microorganisms can colonize the colonic mucosa, which microbial species become dominant, and how microbial signals are presented to epithelial, stromal, and immune cells. Therefore, understanding the CRC microbiome requires a framework that connects microbial ecology with clinically relevant outcomes, including carcinogenesis, immune responsiveness, and therapeutic resistance (Gao R. et al., 2021; Liu et al., 2022; Huang et al., 2023; Zhang et al., 2023; Chen Y. et al., 2025; Bautista et al., 2026; Dong et al., 2026).

Although numerous reviews have explored the relationship between CRC and the gut microbiome, most have been largely bacteria-centric, with limited attention to other microbial kingdoms and, more importantly, their interactions (Wong and Yu, 2023; Shekar et al., 2026). Yet the gut microbiome functions as an interconnected ecological network in which bacteria, fungi, viruses, and other microorganisms continuously interact with the host and another (Gao R. et al., 2021; Liu et al., 2022; Huang et al., 2023; Bautista et al., 2026). These interactions can regulate epithelial barrier function, microbial metabolite production, innate and adaptive immune signaling, microenvironment organization, and the selective pressures imposed by therapy (Gao R. et al., 2021; Liu et al., 2022; Huang et al., 2023; Zhang et al., 2023; Chen Y. et al., 2025; Bautista et al., 2026; Dong et al., 2026). Therefore, examining these interactions in isolation may provide an incomplete understanding of their role in colorectal carcinogenesis, immune regulation, and therapeutic outcome.

Many existing studies have also adopted a primarily taxonomic perspective, focusing on the presence or abundance of specific microorganisms. However, emerging evidence suggests that the effects of many microbial species are highly context and organ-dependent: the same microorganisms implicated in tumor initiation or immune suppression may also influence treatment responsiveness, antitumor immunity, or resistance to therapy through distinct mechanisms (Gur et al., 2015; Gao Y. et al., 2021; Wang X. et al., 2024). Consequently, a purely taxonomy-based framework is insufficient to capture the complexity of host-microbiome interactions in CRC. Greater emphasis should instead be placed on the mechanisms through which microbial communities contribute to tumorigenesis, immune modulation, therapeutic response, and resistance (Yu et al., 2017; Jiang et al., 2023; Wang X. et al., 2024).

This review is also written at a time of significant scrutiny in cancer microbiome literature. The rapid accumulation of multi-kingdom and mechanistic data since 2024 has coincided with a methodological debate over the reliability of some cancer microbiome findings: a landmark pan-cancer microbiome study was ultimately retracted in 2024, while a downstream study was retracted in 2025, disputing the biological relevance of low-biomass intratumoral microbial signals (Gihawi et al., 2023; Poore et al., 2024; Sepich-Poore et al., 2024; Ge et al., 2025; Hermida et al., 2025). A synthesis that does not engage with this debate risks overinterpreting fragile signals, whereas a contamination-aware synthesis can use reproducibility as a sorting principle.

This review has two aims. First, it integrates the bacteriome, mycobiome, and phageome of CRC into a single mechanistic output organized around interkingdom interaction as the functional unit (Gao R. et al., 2021; Liu et al., 2022; Huang et al., 2023; Bautista et al., 2026). In this framework, the clinically relevant entity is often not an isolated organism, but a configured cross-kingdom interaction network whose output depends on local host physiology (Gao R. et al., 2021; Liu et al., 2022; Huang et al., 2023; Zhang et al., 2023; Chen Y. et al., 2025; Bautista et al., 2026; Dong et al., 2026). Second, it interprets the evidence through the recent reproducibility debate in cancer microbiome research, distinguishing robust fecal and mechanistic signals from lower-level intratumoral observations (Breitwieser et al., 2019; Steinegger and Salzberg, 2020; Poore et al., 2024; Hermida et al., 2025; Wang et al., 2025). We therefore begin from the colorectal tumor microenvironment as a physiological and ecological niche, through the individual microbial kingdoms, their interactions, multi-omics approaches, therapeutic consequences, methodological limitations, and future translational directions.

### 1.1 Literature search and evidence appraisal

The literature included in this narrative review was identified through targeted searches of PubMed/MEDLINE and manual screening of the reference lists of relevant primary studies and recent reviews. Searches covered studies indexed up to May 15, 2026, without a predefined lower date restriction. Search concepts combined “colorectal cancer” or “colon cancer” with terms related to the microbiome, microbiota, bacteriome, mycobiome, fungi, virome, phageome, bacteriophages, interkingdom or multi-kingdom interactions, epithelial-barrier dysfunction, inflammation, immunity, metabolism, biofilms, chemotherapy, radiotherapy, immunotherapy, therapeutic resistance, and fecal microbiota transplantation. We focused on English-language, peer-reviewed human CRC studies and CRC-relevant experimental models. Studies involving other tumor types were included only when they provided directly relevant mechanistic or methodological evidence. Trial-registry records were used exclusively to describe ongoing studies and were not considered peer-reviewed clinical evidence.

Primary studies were prioritized over reviews for factual, mechanistic, and clinical claims, whereas reviews were used primarily to provide context and identify additional relevant studies. In interpreting the evidence, we considered study design, replication across independent cohorts, experimental validation, and methodological limitations, particularly contamination and host-read misclassification in low-biomass studies. Throughout the review, we distinguish observational human associations from mechanisms demonstrated in experimental models and from clinically validated effects. The terms “robust, “ “fragile, “ and “contested, “ where used, are intended as descriptive categories reflecting the consistency and methodological support of the available evidence rather than as a formal evidence-grading system.

## 2 The colorectal tumor microenvironment as a multi-kingdom ecological niche

Before considering the individual microbial kingdoms that constitute the CRC-associated microbiome, it is necessary to define the tumor-associated niche in which these cellular communities reside. The central argument of this review is that the clinically relevant unit of the CRC microbiome is often not a single organism, but an interaction community shaped by the local tumor environment. This is not a passive backdrop against which microbes act, but rather an active ecological filter shaped by epithelial barrier integrity, inflammatory signaling, immune-cell organization, oxygen tension, nutrient availability, and metabolite flux. These features determine which organisms can colonize, the metabolic strategies that are favored, and how microbial signals reach epithelial, stromal, and immune cells. In this section, we describe this niche through four interacting axes: epithelial barrier dysfunction, inflammatory signaling, immune remodeling, and metabolic reprogramming.

### 2.1 Epithelial barrier dysfunction and the creation of a colonizable surface

The intact colonic epithelium and its bilayer mucus structure maintain spatial separation between host tissue and the dense luminal microbiome. During colorectal tumorigenesis, this barrier is compromised through disruption of mucus architecture, loss of tight-junction integrity, and emergence of atypical, hypoxic, or necrotic tumor surfaces that differ substantially from healthy mucosa (Zhang et al., 2023; Chen Y. et al., 2025; Dong et al., 2026). These alterations have two principal ecological consequences. First, barrier disruption abolishes the spatial segregation between microorganisms and host tissues, enabling direct microbial interactions with epithelial and stromal cells and facilitating tissue invasion (Dejea et al., 2014). The resulting microbial translocation and sustained exposure to microbial-associated molecular patterns (MAMPs) drive chronic inflammation and persistent immune activation, both defining features of the colorectal tumor microenvironment (Grivennikov et al., 2012). Second, the disrupted mucosal surface provides a favorable niche for the establishment of polymicrobial biofilms (Dejea et al., 2014; Kushwaha et al., 2025). These can incorporate fungal and bacterial members, representing a structural form of interkingdom organization, interaction and source of treatment resistance within the tumor niche (Liu et al., 2022; Galloway-Peña et al., 2024). Clinically, this loss of compartmentalization increases the opportunity for microbial products and organized biofilms to shape inflammation, immune exclusion, and treatment responsiveness.

### 2.2 The inflammatory milieu

In the context of epithelial barrier disruption, inflammation plays a dual role, acting both as a driver of further degradation and as a consequence of barrier dysfunction. Microbial products that cross the compromised epithelium engage pattern-recognition receptors, Toll-like and nucleotide-binding oligomerization domain (NOD)-like receptors, on epithelial and immune cells, activating nuclear factor kappa B (NF-κB) and inflammasome signaling. Subsequently, this drives the production of pro-inflammatory mediators including IL-6, IL-17, TNF-α and IL-1β (Sun and Wang, 2026; Zhang X. et al., 2025; Li et al., 2026a). This bidirectional relationship establishes a positive-feedback loop in which inflammatory signaling further compromises epithelial integrity, facilitating microbial translocation and recruitment of immunosuppressive myeloid populations (Wang K. et al., 2024; Han et al., 2025; Zhang X. et al., 2025). The resulting microenvironment is characterized by profound metabolic and immunological alterations that sustains chronic inflammation and creates selective pressures favoring the expansion of specific microbial taxa (Winter and Bäumler, 2023; Bautista et al., 2026). Consequently, inflammation should be viewed not only as a downstream consequence of barrier disruption, but as a key ecological force shaping the composition and function of the tumor-associated microbiome (Winter and Bäumler, 2023). From a multi-kingdom perspective, inflammation cannot be attributed to a single microbial group. Bacterial, fungal, and viral constituents of the tumor microbiome converge on many of the same pattern-recognition receptors and downstream cytokine networks, collectively shaping the inflammatory landscape of the tumor microenvironment (Tang et al., 2015; Li et al., 2019; Patin et al., 2019; Iliev and Cadwell, 2021). Thus, the inflammatory phenotype of CRC should be regarded as an emergent property of the microbial community rather than the hallmark of any individual kingdom. This functional convergence complicates efforts to identify specific immunological characteristics of a tumor to a single microorganism and instead highlights the importance of considering community-level interactions when interpreting host-microbe relationships in CRC (Liu et al., 2022; Huang et al., 2023). In conclusion, inflammation serves simultaneously as a consequence of barrier disruption and a selective pressure on the tumor-associated microbiome.

### 2.3 The immune landscape

The CRC tumor microenvironment contains diverse immune-cell populations whose balance shapes clinical behavior and therapeutic responsiveness. Regulatory T cells, M2-polarized tumor-associated macrophages, and myeloid-derived suppressor cells are generally associated with immune suppression and poor prognosis, whereas cytotoxic CD8+ T cells and Th1-polarized CD4+ T cells are linked to tumor control and improved response to immunotherapy (Pagès et al., 2018). Tumor genotype alone does not determine the immune landscape, while microbial products and metabolites can shape macrophage polarization, dendritic-cell maturation, T-cell function, and recruitment of immunosuppressive myeloid populations. This regulation is not exclusive to bacteria, because fungal and viral signals can also converge on overlapping innate-immune and cytokine pathways. Microsatellite status further modifies this host-microbial immune interface, with dMMR/MSI-H tumors are typically immune-rich and responsive to checkpoint blockade, whereas most MSS tumors remain relatively immune-cold (Andre et al., 2020). This provides a clinically relevant bridge between microbiome biology and therapeutic response, because microbial communities may influence tumor initiation and the immune context in which treatment is delivered.

### 2.4 Metabolic environment

The metabolic environment is the point at which barrier disruption, inflammation, immune remodeling, and microbial ecology become functionally integrated. Tumor-derived metabolites induce regional hypoxia and lactate accumulation, leading to a dysbiotic community that disrupts the balance of key chemicals (Colegio et al., 2014), including short-chain fatty acids, secondary bile acids, hydrogen sulfide, and nitrogenous metabolites. These metabolites influence host cells and neighboring microbes, shaping tumor growth, immune tone, and microbial community structure (Attene-Ramos et al., 2006; Cao et al., 2017). Butyrate exemplifies the context-dependent that pervades microbial metabolite biology. In certain settings, it functions as an energy source and a histone-deacetylase inhibitor, exhibiting tumor-suppressive and immunomodulatory properties (Donohoe et al., 2011). Conversely, in other contexts, it can lead to resistance (Belcheva et al., 2014). The metabolic environment also serves as a pathway for cross-feeding: one organism can produce a metabolite that is utilized by another, resulting in a metabolite pool that is simultaneously a factor that contributes to its formation (Hertel et al., 2021). A hypoxic, lactate-rich, bile-acid-altered niche is conducive to the growth of specialized bacterial and fungal physiologies, thereby playing a role in determining the nature of interkingdom communications. This demonstrates that the metabolic environment is not merely a consequence of dysbiosis but an active determinant of its development.

**Figure 1:**
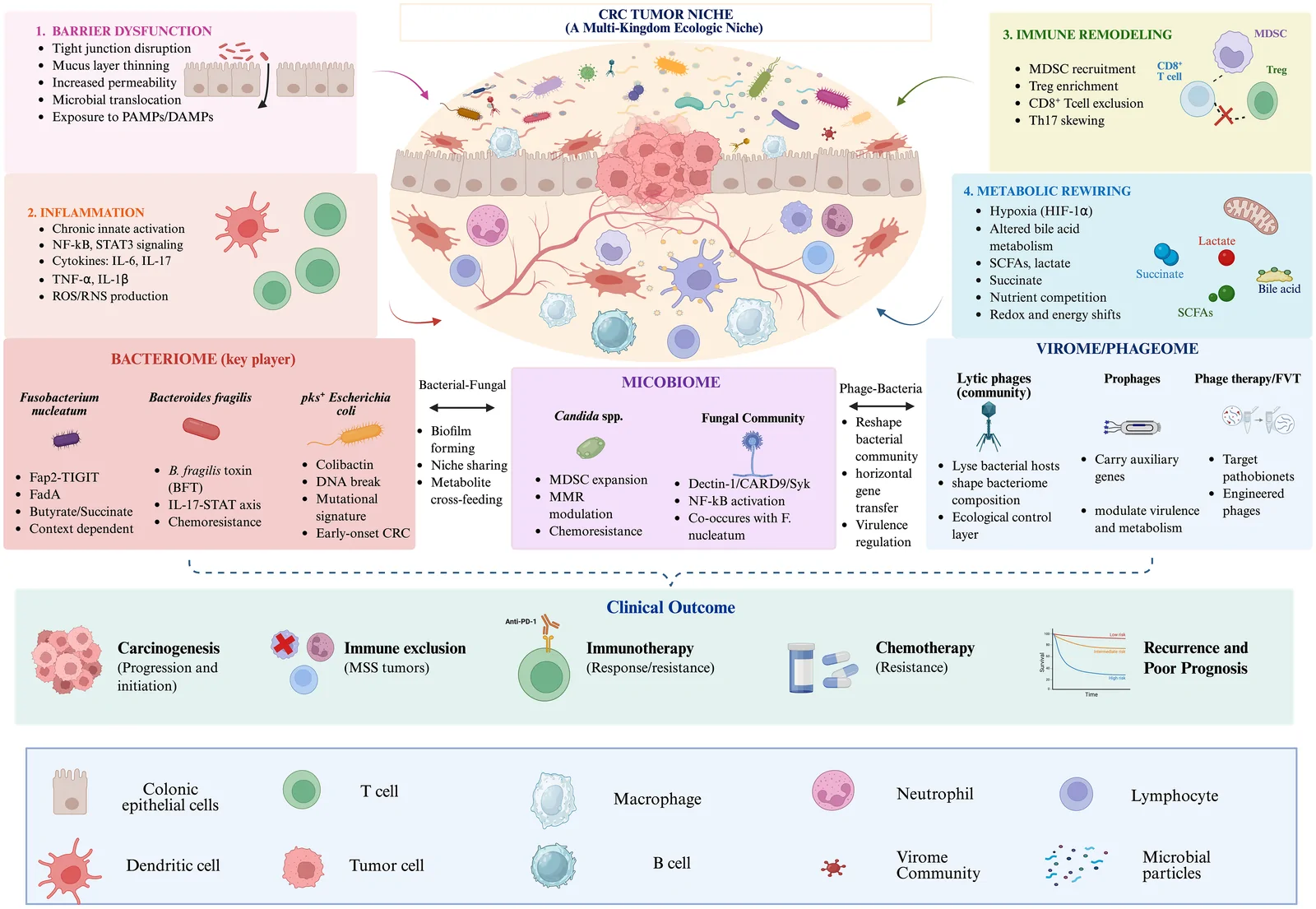
The colorectal tumor niche as a multi-kingdom ecological system integrating barrier dysfunction, inflammation, immune remodeling, and metabolic rewiring. Disruption of epithelial barrier integrity permits microbial translocation and exposure to microbial and damage-associated molecular patterns, creating a permissive surface for microbial colonization and biofilm formation. Chronic inflammatory signaling through NF-κB, STAT3, and cytokine networks reshapes the local microenvironment, while immune remodeling promotes recruitment of myeloid-derived suppressor cells (MDSCs), regulatory T cells, and other immunosuppressive populations. Concurrent metabolic rewiring, characterized by hypoxia, altered bile-acid metabolism, short-chain fatty acids (SCFAs), lactate, succinate, and nutrient competition, further selects for specialized microbial communities. Within this niche, bacterial, fungal, and viral/phage communities interact through biofilm formation, metabolite exchange, ecological competition, and immune regulation. Representative bacterial taxa include Fusobacterium nucleatum, enterotoxigenic Bacteroides fragilis, and pks^+^ Escherichia coli; fungal communities are represented by Candida spp. and other CRC-associated fungi; and bacteriophages modulate bacterial abundance, virulence, and horizontal gene transfer. These interkingdom interactions collectively influence colorectal carcinogenesis, immune exclusion, therapeutic response, chemoresistance, and clinical outcome. The figure illustrates the central concept of this review that the functional unit of the colorectal cancer microbiome is often not an individual microorganism, but an interacting multi-kingdom ecosystem shaped by host physiology. CRC, colorectal cancer; PAMPs, pathogen-associated molecular patterns; DAMPs, damage-associated molecular patterns; NF-κB, nuclear factor kappa B; STAT3, signal transducer and activator of transcription 3; IL, interleukin; TNF-α, tumor necrosis factor alpha; ROS, reactive oxygen species; RNS, reactive nitrogen species; MDSCs, myeloid-derived suppressor cells; Treg, regulatory T cell; Th17, T helper 17 cell; HIF-1α, hypoxia-inducible factor 1 alpha; SCFAs, short-chain fatty acids; TIGIT, T-cell immunoreceptor with immunoglobulin and ITIM domains; FadA, Fusobacterium adhesin A; BFT, Bacteroides fragilis toxin; pks, polyketide synthase; MMR, mismatch repair; Dectin-1, dendritic cell-associated C-type lectin-1; CARD9, caspase recruitment domain-containing protein 9; Syk, spleen tyrosine kinase; FVT, fecal virome transplantation; MSS, microsatellite stable; PD-1, programmed cell death protein 1.

Taken together, these four axes describe a habitat rather than a backdrop. The disrupted barrier establishes a colonizable surface, while inflammation supplies a self-reinforcing signaling setting. This immune landscape is configured in part by microbial input and the metabolic environment both reflects and structures the community. Notably, none of these conditions is specific to a single kingdom, but it is rather a shared determinant that bacteria, fungi and viruses respond and contribute to. It is this niche that makes interkingdom interaction not a special case but the expected mode of organization within which the individual kingdoms and their interactions should be framed. This framework is clinically important because the same niche of microbial colonization may also shape immune responsiveness, resistance to therapy, and the efficacy of microbiome-directed interventions. The conceptual framework proposed throughout this review is summarized in **Figure 1**, which illustrates how barrier dysfunction, inflammation, immune remodeling, and metabolic rewiring generate a multi-kingdom ecological niche that supports cross-kingdom microbial interactions and ultimately influences therapeutic response and disease outcome.

### 2.5 Clinical and host heterogeneity

Microbiome findings in CRC must be interpreted in the context of substantial clinical and host heterogeneity. MSI-H/dMMR and MSS/pMMR tumors differ in immune context and checkpoint-inhibitor sensitivity, while proximal, distal, and rectal tumors differ in anatomy, treatment exposure, and microbial composition (Pagès et al., 2018; Thomas et al., 2019; Wirbel et al., 2019; Andre et al., 2020; Li T. et al., 2025). Disease stage, primary-versus-metastatic sampling, treatment line, surgery, systemic therapy, pelvic radiotherapy, antibiotic or proton-pump inhibitor exposure, bowel preparation, diet, geography, age, and metabolic comorbidities may further modify microbial communities or host responses. Stool, mucosal, primary-tumor, metastatic-tissue, and blood samples should not be considered interchangeable biological compartments. Candidate microbial mechanisms and biomarkers should therefore be evaluated within clinically defined populations and stratified by tumor site, stage, MMR/MSI status, treatment context, sample compartment, and major host exposures. Longitudinal within-patient sampling is particularly important when inferring treatment-associated ecological change (Coker et al., 2019; Thomas et al., 2019; Yachida et al., 2019; Li T. et al., 2025).

## 3 The bacteriome: established clinical mechanisms and recent refinements

The bacteriome is the most extensively studied component of the CRC microbiome and remains the microbial kingdom with the most reproducible evidence across independent cohorts (Thomas et al., 2019; Wirbel et al., 2019; Lin et al., 2025). In this review, bacterial data are treated as the clinical and mechanistic foundation on which a CRC multi-kingdom framework can be built. Three facts justify revisiting this well-studied topic. First, fecal bacterial signatures in CRC have been replicated across cohorts, providing a comparatively robust evidence base (Thomas et al., 2019; Wirbel et al., 2019; Lin et al., 2025). Second, recent work has refined earlier taxonomic associations, recognizing that *Fusobacterium nucleatum* as a distinct driver of oncogenic potential (Zepeda-Rivera et al., 2024). Third, bacterial mechanisms illustrate the limits of taxon-centric interpretation: the same organism may exert opposing effects depending on strain, metabolite output, tumor genotype, immune context, and microbial partners. This section is willing to outline established bacterial mechanisms and recent clinically relevant developments.

### 3.1 Bacterial genotoxins and genomic instability

The most direct mechanism by which gut bacteria contribute to colorectal carcinogenesis is the production of genotoxins that damage host DNA. The best-characterized is colibactin, a hybrid non-ribosomal peptide-polyketide product of the pks genomic island carried by certain *Escherichia coli* and related *Enterobacteriaceae*. This, in turn, alkylates DNA and generates interstrand crosslinks and double-strand breaks (Nougayrède et al., 2006; Bossuet-Greif et al., 2018; Wilson et al., 2019). Exposure to pks-positive *E. coli* generates SBS-pks and ID-pks mutational signatures in human intestinal organoids, establishing causality within that experimental model, whereas concordant signatures in human CRC genomes represent an association compatible with prior exposure (Dziubańska-Kusibab et al., 2020; Pleguezuelos-Manzano et al., 2020). Other bacterial genotoxins broaden this mutagenic spectrum: the *Bacteroides fragilis* toxin (BFT) of *enterotoxigenic B. fragilis* (ETBF) cleaves E-cadherin and activates Wnt/β-catenin and NF-κB signaling (Chung et al., 2018), while cytolethal distending toxin, produced by several Gram-negative intestinal bacteria, exerts genotoxic activity through DNase-mediated DNA damage, promoting replication stress and genomic instability (Tremblay et al., 2021). A recent comprehensive multinational analysis of early onset CRC genomes revealed a significant enrichment of colibactin-associated mutational signatures in these tumors compared to older patients. Furthermore, colibactin-related mutations frequently impact well-established driver genes (Severino and Ianiro, 2025). In light of findings, colibactin-mediated damage can accumulate during early-life colonization, connecting a bacterial genotoxin as a potential candidate for early-life exposure associated with the epidemiological shift towards younger-onset disease. This is supported by mutational-signature epidemiology rather than a proven causal pathway in humans.

### 3.2 Immune modulation, the *Fusobacterium nucleatum* paradox, and clade refinement

Beyond direct genotoxicity, bacteria shape the tumor by modulating local immunity. For instance, *F. nucleatum* has been the principal organism of scientific literature. Recent findings, propose that it suppresses antitumor immunity through several routes: the Fap2 adhesin engages the inhibitory receptor TIGIT on natural killer and T cells, dampening their cytotoxic activity (Gur et al., 2015); FadA-mediated binding to E-cadherin activates β-catenin signaling (Rubinstein et al., 2013), while the organism recruits myeloid-derived suppressor cells and promotes a tolerogenic myeloid compartment (Kostic et al., 2013).

Two recent studies have changed how this organism should be interpreted. First*, F. nucleatum* should no longer be treated as a single biological entity. Closed-genome analysis has shown that *F. nucleatum* subsp. *animalis* separates into distinct clades, with *Fna C2* enriched in the CRC tumor niche and associated with enhanced virulence and metabolic features. This clade-level resolution may explain some of the heterogeneity in earlier studies that treated *F. nucleatum* as a single organism (Zepeda-Rivera et al., 2024). Second, other studies suggest that in MSS CRC*, F. nucleatum* may enhance response to anti-PD-1 therapy. This effect is achieved through the production of bacterial butyrate, inhibition of host histone deacetylases, and a TBX21-dependent pathway that restricts CD8^+^ T-cell exhaustion (Wang X. et al., 2024). This serves as an example of how the same organism can function as an immunosuppressive pathobiont and a tumor suppressor depending on the context.

We deliberately leave this apparent contradiction unresolved here, because the interaction-centered, context-dependent framing is outlined in later sections of this article. The point to carry forward is that the immunological effect of *F. nucleatum* cannot be read off its presence alone; it depends on clade, on the dominant metabolite, on the microsatellite status of the tumor, and on the surrounding community. This dependence provides an early and clinically relevant example of why taxonomic presence alone is insufficient.

### 3.3 Metabolic rewiring of the microenvironment

Bacterial metabolites can exert either tumor-suppressive or tumor-promoting effects depending on their concentration, tissue context, and the broader microbial community. Loss of butyrate-producing bacteria, such as *Faecalibacterium prausnitzii* and *Roseburia*, reduce local butyrate levels, a metabolite that fuels colonocytes, reinforces the epithelial barrier and exerts anti-inflammatory and HDAC-inhibitory effects (Donohoe et al., 2011; Zhou et al., 2018; Faden, 2022). Conversely, dysbiotic communities may favor the production of secondary bile acids such as deoxycholic and lithocholic acid (Bernstein et al., 2005; Májer et al., 2014; Li et al., 2026b), hydrogen sulfide from sulfate-reducing bacteria (Attene-Ramos et al., 2006; Wolf et al., 2022), and polyamines (Goodwin et al., 2011), which can promote cell proliferation, oxidative stress, and a mutagenic milieu. Butyrate itself illustrates the context-dependence that is a the central theme through this review: tumor-suppressive in many settings, and pro-tumorigenic effects under specific other conditions (Belcheva et al., 2014; Sebastian and Mostoslavsky, 2014).

### 3.4 Bacterial drivers of early-onset colorectal cancer

Early-onset CRC, generally defined as CRC diagnosed before age 45, has emerged as growing clinical and epidemiological concern. Its rising incidence cannot be fully explained by germline predisposition alone, strengthening interest in early-life environmental and host-microbial exposures. The bacteriome offers one of the more mechanistically concrete hypotheses for this trend. As noted above, colibactin-associated mutational signatures are enriched in early-onset tumors, supporting a model in which exposure to *pks-positive Enterobacteriaceae* during a formative window of microbial colonization may contribute to a mutational burden that becomes clinically evident decades later (Severino and Ianiro, 2025). Geographic variation in mutational processes further suggests that region-specific microbial or dietary exposures may shape the early-onset CRC landscape. This remains a hypothesis rather than an established causal pathway, with investigation in clinical trials still needed. The relevance of early-onset CRC in this review is therefore not epidemiological alone but rather illustrates how microbial exposures may leave durable genomic and tumor-biological consequences that later influence CRC development.

## 4 The mycobiome: fungal regulation of tumor immunity and therapy response

Evidence linking the mycobiome to CRC is heterogeneous in methodology and evidentiary strength, with this review considering its major components separately. Fecal mycobiome signatures derived from internal-transcribed-spacer (ITS) sequencing (Coker et al., 2019; Lin et al., 2022), together with mechanistically validated fungal immune pathways such as the Dectin-1/CARD9 axis, are supported by biomass-adequate sampling, robust reproducible observations across cohorts (Malik et al., 2018; Wang et al., 2018; Tang et al., 2023). By contrast, low-biomass intratumoral fungal signals, which attracted considerable attention following reports of fungal enrichment within tumor tissues, remain more vulnerable to contamination, reagent-derived background signal, and taxonomic misclassification, and therefore currently warrant a lower level of evidentiary confidence (Dohlman et al., 2021; Dohlman et al., 2022; Narunsky-Haziza et al., 2022; Salzberg et al., 2026). Maintaining this distinction is essential: fecal and mechanistically validated fungal signals support biological inference, whereas low-biomass intratumoral fungal findings require contamination-aware validation since they can be used as biomarkers or therapeutic targets.

### 4.1 Fungal dysbiosis in the colorectal niche

Although the colonic mycobiome is far less abundant compared to the bacteriome, it undergoes consistent modifications in CRC. A consistently reported community-level observations is a shift in the *Basidiomycota*-to-*Ascomycota* ratio, accompanied by expansion of opportunistic genera and loss of mycobiome diversity in carcinoma relative to neighboring mucosa and healthy controls (Coker et al., 2019). Fecal studies have frequently reported enrichment of *Candida*, *Malassezia*, and *Aspergillus* species in CRC cohorts, although the magnitude and consistency of these signals vary across populations and methods (Szklenarik et al., 2024). Several taxa have been proposed as diagnostic companions to bacterial signatures. *Aspergillus rambellii* and other fungal taxa have been proposed as such, but their abundance varies substantially across individuals and populations (Lin et al., 2022).

A distinct line of research focuses on intratumoral fungi and their association with host driver-gene status. Recent analyses have linked specific fungal colonization patterns to *KRAS*-mutant tumor biology, raising the possibility that fungal communities are not merely passengers but co-vary with the oncogenic genotype of the epithelium they inhabit (Yuan et al., 2025). These intratumoral observations are mechanistically appealing, but their clinical validation is often ambiguous. At such low biomass, fungal reads are easily faked: stray DNA from lab reagents, as identified by 18S rRNA amplicon sequencing, and human reads can be confused (Salzberg et al., 2026).

### 4.2 Fungal immune signaling and myeloid regulation

The strongest fungal evidence in CRC is mechanistic rather than purely taxonomic. Fungal cell-wall components, principally β-glucans, mannans, and chitin, are recognized by host C-type lectin receptors, with Dectin-1 (encoded by *CLEC7A*) being the central sensor. Ligation of Dectin-1 recruits spleen tyrosine kinase (SYK), mediate signals through the CARD9-BCL10-MALT1 complex to activate NF-κB and promote inflammasome assembly (Gross et al., 2006; Taylor et al., 2007; Brown et al., 2018). The CARD9 axis is functionally validated in CRC. CARD9 supports antifungal immunity and, through its effects on myeloid cells, shapes fungal response as tumor-restraining or tumor-promoting (Malik et al., 2018; Wang et al., 2018; Tang et al., 2023). This receptor-level circuitry, rather than any single fungal taxon, is the most reproducible fungal contribution to CRC immunobiology.

*Candida albicans* provides the best-characterized effector example. Its secreted peptide toxin candidalysin damages epithelial membranes and drives macrophage pyroptosis. This lytic, inflammatory myeloid cell death has been shown to foster an immunosuppressive tumor microenvironment in CRC. Consistent with a causal role, a candidalysin-neutralizing peptide protects macrophages from pyroptosis and reprograms their metabolic and immune state (Yan et al., 2025). Fungal β-glucan exposure can, by contrast, repolarize tumor-associated macrophages from an immunosuppressive M2-like state toward an M1-like, pro-inflammatory phenotype in a Dectin-1-dependent manner, illustrating that fungal signals are not uniformly pro-tumor but conditioned by receptor context and dose (Liu et al., 2015). On the suppressive side, *Candida tropicalis* promotes the accumulation and immunosuppressive function of myeloid-derived suppressor cells (MDSCs), with NLRP3 inflammasome-derived IL-1β implicated as a key relay between fungal sensing and myeloid suppression (Zhang et al., 2022; Zhang et al., 2024). The net immunological effect of the mycobiome is therefore bidirectional and depends on which fungal species dominates, the receptor engaged, and the metabolic state of the surrounding tissue.

### 4.3 Fungal-bacterial cooperation

Fungi rarely act in isolation. A recurring theme is that fungal and bacterial members of the CRC ecosystem can cooperate physically and metabolically. Fungi contribute structural scaffolding to polymicrobial biofilms, and fungal hyphae may provide adhesion surfaces and protective microenvironments for bacterial partners. However, many of these mechanisms have been characterized largely in oral and other mucosal biofilms, with direct evidence in CRC microenvironment remains limited (Lohse et al., 2018; Ponde et al., 2021). Commensal bacteria, in turn, can constrain fungal overgrowth by producing antifungal metabolites and engaging host immune pathways. This way the fungal community established is shaped by cross-kingdom interaction rather than autonomous fungal expansion (Fan et al., 2015; Jensen et al., 2024). Inflammatory synergy between fungal and bacterial signals can amplify IL-17/IL-22 signaling beyond what either kingdom achieves alone, providing a mechanistic basis for treating the consortium, rather than the species, as the operative unit (Swidergall and LeibundGut-Landmann, 2022).

### 4.4 Mycobiome and therapy response

The mycobiome has emerging clinical relevance as a potential contributor to therapy response, particularly in immunotherapy and chemotherapy. A 2023 pan-cancer analysis reported that fecal fungal composition was associated with response to immune-checkpoint inhibition. Fungal features could identify clinical responders with performance comparable to, or in some analyses exceeding, bacterial features (Huang et al., 2023). The therapeutic relevance of fungi may also extend to chemotherapy. *Candida tropicalis* has been linked to chemoresistance through a lactate-dependent route that intersects with mismatch-repair function, tying a fungal metabolite to the same DNA-repair machinery that influences immunotherapy efficacy (Qu et al., 2021). These findings suggest that fungal signals may carry clinically relevance beyond bacterial profiling alone. However, these reports should be considered early and hypothesis-generating rather than practice-changing, since prospective validation and mechanistic confirmation in CRC-specific treatment cohorts is currently lacking.

## 5 The virome and phageome: emerging regulators of microbial ecology

Among the three microbial kingdoms considered in this review, clinical data for virome are currently limited and therefore discussed as an emerging component of CRC biology. Direct data linking the CRC virome to immune regulation, therapeutic response, or disease progression remain sparse, since virome characterization is methodologically challenging due to the absence of a universal phylogenetic marker, incomplete reference databases, and substantial dependence on computational inference (Shkoporov and Hill, 2019; Li et al., 2022). In addition, viral-like-particle (VLP) enrichment and low-biomass sequencing approaches are susceptible to contamination, amplification bias, and taxonomic uncertainty (Roux et al., 2016; Anne Hallowell et al., 2026). Thus, the studies discussed in this section are interpreted as mechanistically plausible and hypothesis-generating. Consistent with the framework adopted throughout this review, virome-associated findings are weighted by the strength of the underlying evidence rather by novelty. Accordingly, the virome is presented here primarily as a regulator of microbial community structure and therapeutic possibility, and not as a fully validated independent driver of CRC biology.

### 5.1 Enteric virome dysbiosis

The human gut virome is dominated numerically by bacteriophages, with a smaller eukaryotic-virus component. Metagenomic surveys report CRC-associated shifts in viral community composition, typically enrichment of *Microviridae* and small genome phages, with less consistent changes among *Siphoviridae* and *Myoviridae*. These alterations are detectable as early as the adenoma stages (Hannigan et al., 2018; Nakatsu et al., 2018). Several analyses have proposed virome-derived diagnostic signatures and altered functional content in CRC-associated viral communities. However, whether these changes directly influence epithelial or immune biology, or instead track underlying bacterial dysbiosis, remains unresolved (Zuo et al., 2022). Eukaryotic viruses have also been examined for direct oncogenic contributions, with their role in CRC being less defined than in virally driven malignancies elsewhere in the gastrointestinal tract (Li et al., 2023).

### 5.2 Phage regulation of bacterial virulence

The best characterized viral contribution to CRC is indirect, including the phage-mediated control of the bacteriome. Temperate phages integrate into bacterial chromosomes as prophages and can carry auxiliary metabolic genes (AMGs) and virulence determinants. These, in turn, mediate horizontal gene transfer (HGT), reshaping the functional capacity of their hosts (Shkoporov and Hill, 2019). A recent report identified CRC-specific prophages within *Bacteroides fragilis* populations, providing an example of how the viral compartment may modulate the virulence of a clinically important pathobiont rather than acting on the epithelium directly (Yang et al., 2025; Damgaard et al., 2026). Given that phages set the abundance and gene content of their bacterial hosts, the phageome is best characterized as a regulatory layer sitting above the bacteriome.

A complementary line of evidence comes from a murine model in which *Helicobacter pylori* infection of Apc^+/1638N^ mice was accompanied by an early-stage expansion of temperate phages, including phages predicted to infect CRC-associated bacteria such as *Enterococcus faecalis*. Co-occurrence-network analysis revealed strong viral-bacterial associations arising before tumor development, suggesting that prophage activation and subsequent phage proliferation may favor carcinogenesis indirectly, by reshaping the bacterial profiling rather than by acting directly on the epithelium (Luo et al., 2023). Although still early and largely correlative, these data support the concept that the phageome may act as a regulatory layer of the bacteriome by shaping bacterial abundance, gene content, and pathobiont behavior.

### 5.3 Virome-immune interactions

Phages and eukaryotic viruses present nucleic-acid patterns that engage host pattern-recognition receptors, principally the endosomal Toll-like receptors TLR3 (double-stranded RNA), TLR7 (single-stranded RNA), and TLR9 (unmethylated CpG DNA), with downstream type-I interferon induction (Kawai and Akira, 2010). In principle, this allows the virome to tune mucosal immune tone independently of bacteria, with phage translocation across a compromised epithelial barrier delivering immunostimulatory signals to the lamina propria (Nguyen et al., 2017; Sausset et al., 2020; Carroll-Portillo et al., 2025). Phage barrier-crossing and immune activation have been experimentally demonstrated in intestinal models, but their operation and consequences in the CRC tumor microenvironment remain undocumented. Virome-driven interferon modulation should therefore be considered a plausible, hypothesis-generating mechanism for CRC pathway development.

### 5.4 Phage-mediated therapy response and engineering

At present, the clearest therapeutic relevance of the virome may be as an engineering substrate. The property that makes temperate phages biologically interesting, exquisite host specificity and makes them attractive precision tools for editing the bacteriome. One of the strongest therapeutic proof-of-concept examples in CRC is the use of a lytic phage to eliminate *B. fragilis*. In preclinical models, this intervention restores, with this phage-bacterium intervention demonstrating a causal, directional control over a resistance-associated taxon (Ding X. et al., 2025). Beyond natural phages, engineered approaches are advancing; investigators have combined phage administration with fecal virome transplantation (FVT) to reshape recipient microbiome and engineered bacterial chassis to deliver phage-encoded payloads, including constructs in which engineered *Escherichia coli* deliver an M13 phage carrying a PD-1-directed cargo (Li et al., 2023; Li HR. et al., 2025). These strategies remain in early stage, but they suggest that the most immediate translational value of the virome in CRC may lie less in observational signatures than in programmability. **Table 1** summarizes all the recent data around gut microbiome.

**Table 1:** The multi-kingdom CRC microbiome: representative taxa, mechanisms, and functional relevance.

Taxon	Key factor/metabolite	Mechanism	Effect
BACTERIOME			
*pks^+^ Escherichia coli (*Severino and Ianiro, 2025)	Colibactin (genotoxin)	Alkylates DNA, interstrand crosslinks → double-strand breaks; SBS-pks/ID-pks mutational signatures; enriched in early-onset CRC	Pro-tumorigenic (initiation); links to early-onset disease
*Enterotoxigenic Bacteroides fragilis (*Ding X. et al., 2025)	B. fragilis toxin (BFT)	Cleaves E-cadherin → β-catenin/NF-κB; IL-17-STAT3 inflammatory axis; also drives chemoresistance via SusD/RagB-Notch1-EMT	Pro-tumorigenic; context-dependent chemoresistance
*Fusobacterium nucleatum (Fna C2 clade) (*Wang X. et al., 2024)	Fap2; FadA; butyrate; succinic acid	Fap2-TIGIT immune suppression; FadA-E-cadherin-β-catenin; CRC-adapted clade refinement; context-dependent ICI effect (see Table note)	Dual: immunosuppressive yet MSS-ICI-sensitizing depending on context
*Faecalibacterium prausnitzii/Roseburia (*Donohoe et al., 2011)	Butyrate (SCFA)	HDAC inhibition; colonocyte fuel; barrier reinforcement; anti-inflammatory depleted in CRC	Protective (loss is pro-tumorigenic)
MYCOBIOME			
*Candida tropicalis (*Qu et al., 2021)	Lactate; immune signaling	Promotes MDSC expansion; lactate production modulating mismatch-repair → chemoresistance	Pro-tumorigenic; fungal chemoresistance
*Gut mycobiome (community) (*Huang et al., 2023)	Fungal cell-wall β-glucan	Fungal signatures associate with immune checkpoint inhibitor response; fungi AUC may exceed bacteria for prediction	Predictive of immunotherapy response
VIROME/PHAGEOME			
*Bacteriophages (community) (*Nakatsu et al., 2018)	–	Lyse bacterial hosts; transfer auxiliary metabolic/virulence genes; regulate bacteriome composition (ecological control layer)	Indirect - shape bacterial pathobiont abundance
*Bacteroides fragilis prophage (*Damgaard et al., 2026)	Prophage-encoded factors	CRC-associated prophages alter host-bacterium behavior	Emerging; modifies pathobiont function
*Phage therapy/FVT (intervention) (*Kenneth et al., 2025)	Engineered/lytic phages	Phage VA7 eliminates B. fragilis to restore chemosensitivity; engineered PD-L1-displaying M13 phage	Therapeutic - multi-kingdom modulation
ARCHAEA (acknowledged, not covered in depth)			
*Methanogenic/halophilic archaea (*Li T. et al., 2025)	–	Altered archaeal composition across multi-cohort CRC metagenomes; archaeal-bacterial interactions; diagnostic models	Emerging diagnostic signal

AUC, area under the curve; BFT, *Bacteroides fragilis* toxin; CRC, colorectal cancer; EMT, epithelial–mesenchymal transition; FadA, Fusobacterium adhesin A; Fap2, Fusobacterium autotransporter protein 2; Fna, Fusobacterium nucleatum subsp. animalis; FVT, fecal virome transplantation; HDAC, histone deacetylase; ICI, immune checkpoint inhibitor; ID, insertion–deletion; IL, interleukin; MDSC, myeloid-derived suppressor cell; MSS, microsatellite stable; NF-κB, nuclear factor kappa B; PD-L1, programmed death-ligand 1; pks, polyketide synthase; SBS, single-base substitution; SCFA, short-chain fatty acid; STAT3, signal transducer and activator of transcription 3; TIGIT, T-cell immunoreceptor with immunoglobulin and ITIM domains.

## 6 Interkingdom interaction as the functional unit

Most CRC microbiome reviews are organized around individual organisms or taxonomic groups, typically treating *Fusobacterium nucleatum*, *enterotoxigenic Bacteroides fragilis*, *pks-positive Escherichia coli*, and other candidate pathobionts in isolation. That taxon-centric framing has been promising but faces important clinical limitations: it cannot fully explain why the same species is associated with opposite outcomes across cohorts, why single-microbe biomarkers often generalize poorly, or why community-level signatures frequently outperform individual taxa in prediction (Liu et al., 2022; Wang X. et al., 2024). The central argument of this section is that clinically relevant microbiome effects often emerge from interactions among organisms rather than from the isolated presence of a single taxon. Existing reviews acknowledge cross-kingdom and polymicrobial effects, and some emphasize biofilm-structured polymicrobial consortia as tumor-promoting units (Chen C. et al., 2025; Zhang C. et al., 2025; Bautista et al., 2026; Li et al., 2026a). However, limited reports treat interkingdom interaction as the explicit analytic unit linking microbial ecology to immune modulation, therapeutic response, and resistance.

### 6.1 Bacterial-fungal interactions

The bacterial-fungal interface is the best-developed cross-kingdom axis in CRC. Physically, fungi and bacteria co-assemble into biofilms in which fungal hyphae provide structural scaffolding and adhesion surfaces while bacteria contribute metabolic specialization, producing a spatially organized consortium with oxygen and metabolite gradients that neither kingdom generates alone. Ecologically, the relationship is dynamic: across the healthy to adenoma to carcinoma sequence, fungal-bacterial associations shift from a co-occurrence regime, in which taxa rise and fall together, toward a co-exclusion regime characteristic of a destabilized, competitive community. This is an ecological state-change that parallels the loss of network structure seen in progression (Coker et al., 2019; Yuan et al., 2025).

A particularly important recent advance is a molecularly resolved cross-kingdom mechanism. Work published in early 2026 described a direct, adhesin-mediated interaction between *Candida albicans* and *F. nucleatum*, in which a fungal adhesin engages a fusobacterial outer-membrane adhesin to nucleate a mixed consortium that drives recruitment of myeloid-derived suppressor cells and thereby suppresses antitumor immunity. The crucial aspect is that the interaction is amenable to pharmacological intervention. Specifically, supplying L-arginine disrupts the adhesin interaction that underpins the consortium and reverses its immunosuppressive effect. The human cohort findings are associative, whereas the Flo9–RadD interaction and the effects of L-arginine are supported by mechanistic experiments in organoid and animal models (Li J. et al., 2026; Lobel, 2026). This appears to be among the first molecularly resolved examples in CRC in which a cross-kingdom partnership is defined by the adhesion molecules that hold it together and can be disrupted by a defined intervention. It provides one of the strongest available examples that the interaction, rather than either partner in isolation, can be the operative and therapeutically tractable entity.

### 6.2 Phage-bacteria ecological dynamics

Sitting above the bacteriome, the phageome exerts control over bacterial abundance and gene content. Lytic phages impose density-dependent mortality on susceptible hosts, generating predator-prey oscillations that continuously reshape the bacterial community (Thingstad, 2000), while also altering host phenotype directly through lysogenic conversion and their auxiliary carrier-genes (Shkoporov and Hill, 2019). In CRC, the bacterial taxa available to interact with fungi, epithelium, and immune system are a moving function of phage pressure. Ecological theory predicts that targeting a single bacterial species, by antibiotic or phage, propagates through the community rather than staying on-target: removing a keystone taxon can release its competitors and, via apparent competition, restructure fungal with metabolic relationships several steps being removed (Marantos et al., 2022). While direct evidence in CRC remains limited, a recent report illustrated the reversal of chemoresistance following phage-mediated depletion of *B. fragilis*, supporting that single-taxon editing can produce consequences beyond the intended target (Ding X. et al., 2025). For this reason, phage-based editing is best evaluated for its community-level effects rather than its on-target effect alone. This is clinically important, because a phage strategy that removes one resistance-associated bacterium may still reshape fungal expansion, metabolite pools, and immune tone in unpredictable ways.

### 6.3 Cross-kingdom metabolic cooperation

Metabolism is the common modulator through which kingdoms interact, with several circuits converge on a shared set of hosts signaling nodes in CRC. Bile-acid transformation is a multi-step bacterial relay: primary bile acids are deconjugated and dehydroxylated by sequential bacterial activities into secondary bile acids such as deoxycholic and lithocholic acid, which act as signaling ligands on the epithelium and on immune cells through FXR, TGR5, and Wnt/β-catenin (Furusawa et al., 2013; Cao et al., 2017; Modoux et al., 2022). Short-chain fatty acids, chiefly butyrate, are produced by specialized bacterial guilds from dietary fiber and cross-fed between species, and their local concentration sets epithelial energy status and immune tone, largely through HDAC inhibition (Bernstein et al., 2005; Donohoe et al., 2011). Nitrogen is exchanged in both directions, with L-arginine emerging as a pivotal shared node: exogenous L-arginine disrupts the *Candida-Fusobacterium* partnership, and arginine availability conditions both myeloid suppression and T-cell function (Rodriguez et al., 2004; Li J. et al., 2026). Critically, these inputs are not parallel and kingdom-specific but convergent. Fungal effectors, candidalysin and fungal-derived organic acids among them, feed into the same nodes that bacterial metabolites engage: HIF-1α, the aryl hydrocarbon receptor (AhR), and histone deacetylases (HDAC). These nodes are interlinked, with butyrate-mediated HDAC inhibition potentiates AhR signaling, so that fungal and bacterial signals are integrated rather than additive, and the tumor experiences a single, blended metabolic input (Donohoe et al., 2011; Zhou et al., 2018; Modoux et al., 2022; Zhang et al., 2022). The metabolite layer is thus where cross-kingdom cooperates, being the layer at which the most plausible intervention, such as substrate supplementation, dietary modification, and metabolite mimetics, act.

### 6.4 Cross-kingdom immune co-regulation and the *F. nucleatum* paradox

The clearest demonstration that context overrides identity is the *F. nucleatum* paradox. The same organism is reported to suppress antitumor immunity through Fap2-TIGIT engagement and myeloid recruitment (Kostic et al., 2013; Gur et al., 2015). Yet in microsatellite-stable (MSS) CRC, intratumoral *F. nucleatum* can enhance anti-PD-1 efficacy by supplying butyrate. This, in turn, inhibits histone deacetylases HDAC3 and HDAC8 in CD8^+^ T cells, inducing H3K27 acetylation at the Tbx21 promoter, driving TBX21 (T-bet) up-regulation, leading to reduction of PD-1 expression to produce a more cytotoxic, less exhausted phenotype (Wang X. et al., 2024). Furthermore, in metastatic CRC, *F. nucleatum*-derived succinic acid suppresses the cGAS-interferon-β pathway, lowering the Th1 chemokines CCL5 and CXCL10 and limiting CD8^+^ T-cell trafficking into the tumor, thereby conferring resistance to anti-PD-1 therapy (Jiang et al., 2023). Whether the organism helps or harms therefore depends on which metabolite dominates its output, and on the immune substrate it acts on, variables that are themselves set by its cross-kingdom partners and the surrounding nutrient environment. In a butyrate-permissive context the HDAC-inhibitory, T-cell-sensitizing face predominates; in an arginine-fueled, Candida-partnered, MDSC-recruiting context the immunosuppressive face predominates (Li J. et al., 2026). This apparent contradiction reflects the limits of classifying *Fusobacterium* as inherently beneficial or harmful; its role instead depends on the surrounding microbial and host context. Fungal and viral co-modulators feed into the same circuitry: fungal β-glucan-driven macrophage repolarization (Liu et al., 2015), and phage-driven shifts in interferon tone modulate the HDAC/T-bet, cGAS-IFNβ, and myeloid-suppression axes that, together, shape which fusobacterial face is expressed. This paradox is therefore best interpreted as a clinically relevant switch, in which nutrient availability, microbial partners, metabolite dominance, and immune-cell state determine whether the same organism produces a suppressive or sensitizing output.

### 6.5 Ecological instability and tumor evolution

If interactions are the unit, then carcinogenesis can be read as an ecological trajectory. The healthy colonic ecosystem is a structured network with redundancy and stabilizing negative feedback; dysbiosis in CRC is not just a change in membership but a change in the system’s state, loss of network connectivity (Qian et al., 2025), a shift from cooperative co-occurrence to competitive co-exclusion (Nakatsu et al., 2015; Coker et al., 2019; Mo et al., 2020; Yuan et al., 2025), and diminished diversity with a likely loss of resilience (Bai et al., 2025). Microbial succession along the adenoma to carcinoma sequence accompanies, and plausibly contributes to, the genetic and microenvironmental evolution of the lesion, with early-stage communities differing in structure from those of established carcinoma (Nakatsu et al., 2015; Coker et al., 2019; Mo et al., 2020; Yuan et al., 2025). These cross-sectional observations demonstrate stage-associated differences in community composition and network structure, but they do not establish within-patient ecological-state transitions. We therefore use the term “ecological state” as an organizing and testable interpretation rather than as a directly demonstrated longitudinal process in CRC. This ecosystem-state provides the conceptual bridge to therapeutic resistance, where resistance is recast as a property of community adaptation rather than as the action of any single resistant microbe. **Table 2** summarizes the interkingdom interaction with their corresponding mechanisms.

**Table 2:** Interkingdom interactions shaping CRC biology: the interaction, not the single taxon, as the functional unit.

Interaction type	Representative players	Mechanism of interaction	Functional/immune consequence	Evidence status	Clinical relevance
Bacterial-fungal (Coker et al., 2019; Li J. et al., 2026)	*F. nucleatum + Candida* spp.*; A. rambellii co-occurrence*	Biofilm co-aggregation, shared anaerobic/hypoxic niche, metabolite cross-feeding; shift from co-occurrence (healthy) to co-exclusion (CRC)	MDSC recruitment, Th17 skewing, sustained inflammation	Mixed	Progression; potential resistance
Phage-bacterial (Nakatsu et al., 2018; Damgaard et al., 2026)	*Temperate phages + CRC pathobionts (e.g. B. fragilis prophage)*	Lysis of specific hosts; horizontal gene transfer of virulence/metabolic genes; prophage modulation of host-bacterium behavior	Indirect immune modulation via control of bacterial pathobiont abundance	Emerging	Phage therapy; FVT
Cross-kingdom metabolic (Jiang et al., 2023; Wang X. et al., 2024)	*SCFAs, secondary bile acids, lactate, succinate, fungal metabolites*	Shared metabolic niche; fermentation products of one organism are substrates/signals for another and for host immune cells	CD8^+^ T-cell exhaustion vs activation depending on dominant metabolite (e.g. butyrate vs succinate)	Partly robust	ICI response modulation
Bacterial-fungal-immune (network) (Huang et al., 2023)	*Responder vs non-responder multi-kingdom ecosystems*	Interkingdom bacterial-fungal correlation networks present in ICI responders, effectively absent in non-responders	Ecologic network structure tracks immune competence better than single-taxon abundance	Robust	Predictive biomarker (interaction-level)
Ecologic-state (succession) (Nakatsu et al., 2015; Coker et al., 2019)	*Whole multi-kingdom community across adenoma-carcinoma sequence and under therapy*	Cross-sectional studies demonstrate stage-associated community reorganization; within-patient treatment-selected transitions remain untested	Resistant versus responsive community states are a conceptual interpretation, not a directly demonstrated CRC transition	Framework	Hypothesis for biomarker development and FMT timing

CRC, colorectal cancer; FMT, fecal microbiota transplantation; FVT, fecal virome transplantation; ICI, immune checkpoint inhibitor; MDSC, myeloid-derived suppressor cell; SCFAs, short-chain fatty acids; Th17, T helper 17 cell.

## 7 Multi-omics for resolving cross-kingdom architecture and clinical signal

Multi-omic measurement is essential for moving CRC microbiome research beyond taxonomic cataloguing but toward clinically interpretable mechanisms. Its role is not simply to describe which organisms are present, but to determine whether bacteria, fungi, and phages form spatially organized, metabolically active, and immunologically relevant communities. At the same time, the methods used to detect these communities strongly influence whether the recovered signal is biological or artifactual. This is particularly important in multi-kingdom studies, where each kingdom is measured with different assays, reference databases, and contamination risks.

### 7.1 Per-kingdom sequencing and assay limitations

No single assay captures all three kingdoms adequately. Bacterial communities are profiled by 16S rRNA gene amplicon sequencing (taxonomic, inexpensive, but limited in resolution and functional content) or by broad and inaccurate metagenomics (strain- and function-resolved, but more sensitive to host-DNA contamination at low microbial biomass) (Salter et al., 2014). Fungi require dedicated ITS or 18S rRNA gene sequencing, with reference-database gaps that make fungal taxonomy less reliable than bacterial (Li et al., 2022; Salzberg et al., 2026). Viruses lack any universal marker gene and depend on VLP enrichment followed by shotgun sequencing, with assembly and database limitations that make virome profiling the least standardized of the three (Shkoporov and Hill, 2019). The practical lesson for clinically oriented cross-kingdom work is that reliable multi-kingdom interpretation requires orthogonal assays, explicit acknowledgment of assay-specific biases, and caution when comparing kingdoms profiled with methods of unequal reliability (Faust et al., 2015; Gloor et al., 2017; Brunner et al., 2024).

### 7.2 Metabolomics as the common currency

Because kingdoms interact through metabolites, untargeted and targeted metabolomics provide the most direct read-out of agnostic and cross-kingdom data type available (Moskowitz et al., 2020). Fecal and tissue metabolite profiles [bile acids (Bernstein et al., 2005), SCFAs (Cao et al., 2017), amino acids including arginine, polyamines, and indole derivatives (Li et al., 2026a)] integrate the activity of all community members and connect microbial composition to the host signaling nodes [HDAC (Furusawa et al., 2013), AhR (Modoux et al., 2022), HIF-1α (Zhang et al., 2022)] that govern the tumor response. Pairing metabolomics with sequencing converts a list of co-occurring taxa, converting this into a mechanistic hypothesis about their complex co-dependences. This data type is less susceptible to the low-biomass amplification artifacts that afflict taxonomic sequencing (Yachida et al., 2019; Hertel et al., 2021). Clinically, this makes metabolomics one of the most useful bridges between microbial ecology and treatment-relevant phenotypes, since metabolite outputs can be linked to immune tone, epithelial stress, and resistance pathways more directly than taxonomic abundance alone.

### 7.3 Spatial and single-cell approaches

Interaction is intrinsically spatial: whether a microbe influences a T cell, macrophage, or epithelial cell depends on proximity. Spatial transcriptomics, spatial metabolomics, and *in situ* hybridization for microbial taxa allow microbe-immune and microbe-epithelial relationships to be measured where they occur, while single-cell RNA sequencing resolves the host-cell states induced by microbial signals. An integrative analysis associated a butyrate–HDAC signature with CD8+ T-cell exhaustion and provided partial mechanistic support through *in vitro* butyrate supplementation (Chen X. et al., 2025). By integrating spatial and microbial data, such approaches can localize interkingdom-relevant mechanisms to defined cellular neighborhoods rather than inferring them from bulk averages. The same study derived a spatial microbiome score that stratified tumors by microbial-metabolic dysfunction and predicted immunotherapy resistance, illustrating that spatially resolved interaction data may carry clinical as well as mechanistic value. However, the derived immunotherapy-resistance score was based on computational surrogates and has not been prospectively validated in treated CRC patients (Chen X. et al., 2025).

### 7.4 Network inference and contamination-aware pipelines

Reconstructing interkingdom networks from multi-omic data requires statistical inference, including correlation-based approaches, probabilistic graphical models, and increasingly machine-learning methods. These tools can identify candidate relationships among bacteria, fungi, phages, metabolites, and host immune features, but they are also highly sensitive to noise. If contaminants, host-read misclassification, or reagent-derived background signals are present in the data, network methods may convert them into apparently meaningful biological relationships. The countermeasure is a contamination-aware analytical stack: negative and reagent controls (Salter et al., 2014), in silico decontamination (Davis et al., 2018), biomass-aware thresholds (Salter et al., 2014), and host-read filtering applied before any network is built (Brunner et al., 2024). Treating these controls as integral to the analysis, rather than ancillary steps, is essential for distinguishing genuine cross-kingdom architecture from technical artifact (Salzberg et al., 2026).

## 8 Cross-kingdom regulation of therapeutic response and resistance

### 8.1 Immunotherapy

Mismatch-repair status is the principal determinant of checkpoint inhibitor efficacy in CRC. MSI-H/dMMR tumors, with their high neoantigen burden and inflamed immune microenvironment, are more likely to respond to checkpoint blockade, whereas the much larger MSS/pMMR population is generally immune-cold and largely refractory (Andre et al., 2020; Wang X. et al., 2024). The central therapeutic question is therefore not whether immunotherapy can work in CRC, but how immune-excluded MSS tumors might be shifted toward a responsive state. The microbiome is one of the few modifiable factors that may shape this immune context.

The pivotal mechanistic evidence is the fact that intratumoral *F. nucleatum* can sensitize MSS CRC to anti-PD-1 therapy by supplying butyrate that inhibits HDAC3/8 in CD8+ T cells and upregulates TBX21. This finding establishes a preclinical mechanism but not a clinically validated predictive biomarker (Wang X. et al., 2024). In this setting, a bacterium often associated with tumor progression becomes a potential immunotherapy sensitizer when its metabolic output acts on a permissive immune substrate. This example captures the central theme of this review: therapeutic effect cannot be inferred from microbial presence alone, but depends on the functional output of the surrounding microbial and immune ecosystem.

The case for a multi-kingdom predictive model is also emerging clinically. A recent analysis across more than seventeen hundred samples reported that a multi-kingdom classifier built from 32 genera spanning multiple kingdoms outperformed single-kingdom models for ICI-response prediction (Qiao and Zhu, 2025), while another found that fungal features could discriminate responders from non-responders with performance comparable to, or in some analyses exceeding, bacterial features (Huang et al., 2023). These findings suggest that ignoring non-bacterial kingdoms may discard clinically useful predictive information. However, these models, even though promising, should still be interpreted as early and hypothesis-generating until validated prospectively in CRC-specific immunotherapy cohorts. Microbiome-associated effects on immunotherapy should therefore be evaluated within MMR/MSI status, tumor site, treatment line, and major host exposures rather than treated as independent determinants of response. At present, MMR/MSI status remains the principal established biomarker guiding checkpoint-inhibitor use in CRC (Andre et al., 2020), whereas microbiome signatures remain investigational and are not validated for treatment selection or patient stratification.

### 8.2 Chemotherapy

*F. nucleatum* is one of the potential microbial mediator of chemoresistance, with recent work has shed light on its mechanisms. It has been established that, via an autophagy-dependent mechanism, *Fusobacterium* blunts 5-fluorouracil and oxaliplatin cytotoxicity (Yu et al., 2017), while also induces the upregulation of the anti-apoptotic factor BIRC3 to resist 5-fluorouracil (Zhang et al., 2019). Another study identified a Hippo/YAP-linked pathway in which *F. nucleatum* modulates GSDME-dependent pyroptosis to promote chemoresistance (Wang N. et al., 2024). On the fungal side*, C. tropicalis* has been linked to chemoresistance through a lactate-dependent impairment of mismatch-repair function, tying a fungal metabolite to the same DNA-repair axis that defines immunotherapy eligibility (Qu et al., 2021). Across these examples, chemoresistance emerges through convergent regulation of epithelial stress responses, apoptosis, pyroptosis, autophagy, and DNA-repair competence rather than through a single kingdom-specific pathway. These findings establish mechanisms within the tested experimental models but do not validate either *F. nucleatum* or *Candida tropicalis* as a treatment-selection biomarker in CRC patients.

### 8.3 Radiotherapy

Radiotherapy both perturbs and is modulated by the microbiome. Pelvic irradiation induces dysbiosis that contributes to radiation enteropathy, while specific commensals condition radiosensitivity. In a 2024 study, preclinical data showed that *Roseburia* intestinalis-derived butyrate enhances radiosensitivity in CRC by promoting radiation-induced autophagy through an OR51E1/RALB signaling axis, identifying a defined bacterium-metabolite-host circuit that tunes the radiation response (Dong et al., 2024). Radiotherapy remains the least-developed therapeutic axis in the CRC microbiome literature, and whether fungal or viral members modulate radiosensitivity in comparable ways is largely unexplored. This gap is clinically relevant for rectal cancer, where pelvic radiotherapy directly perturbs the mucosal ecosystem and may select for treatment-adapted microbial states. These findings remain preclinical; prospective evidence linking microbiome features to radiosensitivity in patients with rectal cancer is not yet available.

### 8.4 A proposed treatment-selected ecological-state model of therapeutic resistance

We propose that therapeutic resistance may sometimes be conceptualized as a treatment-selected ecological state; however, direct evidence for such state transitions in CRC is currently limited. *F. nucleatum* illustrates this point since it can produce opposing therapeutic effects across contexts and modalities. It has been linked to chemoresistance through autophagy-dependent mechanisms and Hippo/YAP-GSDME-mediated inhibition of pyroptosis (Wang N. et al., 2024), and to ICI resistance through a succinic-acid route that suppresses the cytosolic DNA-sensing/interferon pathway and limits CD8+ T-cell trafficking (Jiang et al., 2023). Conversely, its butyrate output may favor immunotherapy response by promoting HDAC inhibition in CD8+ T cell) 2), it has also been linked to downregulation of mismatch-repair proteins such as MLH1 and MSH6, potentially shifting tumor immunogenicity (Ding T. et al., 2025). Thus, the clinical effect of a microbe depends less on its presence alone than on the ecological context that determines its dominant metabolic and immune output.

The available evidence comprises three distinct layers. First, clinical and observational studies demonstrate treatment-associated changes in microbial community composition (Yuan et al., 2018; Ding T. et al., 2025). Second, experimental studies identify organism- and metabolite-specific mechanisms through which *F. nucleatum* can modify chemotherapy or anti-PD-1 response (Furusawa et al., 2013; Yu et al., 2017; Zhang et al., 2019; Jiang et al., 2023; Wang X. et al., 2024). Third, the proposition that these changes constitute a coordinated multi-kingdom transition from a treatment-sensitive to a treatment-resistant state represents our conceptual interpretation. No prospective longitudinal study has yet directly demonstrated such a transition in CRC patients.

This ecological view has several clinical implications. First, treatment itself can reshape the microbial ecosystem. Cytotoxic and immune therapies can alter community structure (Yuan et al., 2018; Wan et al., 2022), and treatment-associated community changes may accompany response or resistance, but their mediating role has not been established (Davar et al., 2021). Second, it explains why single-microbe biomarkers often fail to generalize: if resistance is a system property, then any individual taxon is at best a proxy for the broader ecological state. This is consistent with evidence that community-level and interkingdom-network signatures may outperform single-kingdom or single-taxon models (Huang et al., 2023; Qiao and Zhu, 2025). Third, it redirects biomarker development toward ecological-state descriptors, including network connectivity, metabolite-output profiles, diversity and resilience metrics, and spatial proximity between microbes and immune cells.

Finally, this framework makes the timing of microbiome-directed intervention clinically important. Fecal microbiota transplantation and related strategies can be understood as attempts to shift the ecosystem toward a treatment-sensitive state, but their effect may depend on when they are applied relative to chemotherapy, radiotherapy, or immunotherapy (Davar et al., 2021; Huang et al., 2022; Cheng et al., 2023). The therapeutic goal is therefore not simply to remove a “bad” microbe or add a “good” one, but to move the microbial ecosystem toward a state that supports antitumor immunity, preserves epithelial resilience, and limits resistance-promoting metabolic programs.

Moreover, the CRC microbiome should be regarded as a dynamic therapeutic target rather than a static biomarker. Every intervention, including chemotherapy, radiotherapy, immunotherapy, antibiotics, diet, and microbiome-based therapies has the potential to reshape ecological interactions, creating both opportunities and new selective pressures.

This framework generates three testable predictions: longitudinal within-patient sampling should identify reproducible changes in community connectivity, metabolite output, or spatial organization before or during acquired resistance; community-level descriptors should add predictive value beyond tumor genotype and individual taxon abundance; and ecosystem-directed interventions should produce measurable functional changes that mediate treatment response. These predictions require prospective validation in clinically defined CRC cohorts.

## 9 Methodological reckoning: what is supported from the evidence

### 9.1 The contamination problem

The clinical promise of the CRC microbiome depends on distinguishing robust biological signal from technical artifact. This distinction is especially important for multi-kingdom and intratumoral microbiome studies, because tumor tissue and blood often contain very low microbial biomass relative to host DNA. At low biomass, three sources of artifact can dominate: reagent and laboratory contamination, host-read misclassification, and batch or sequencing-center effects that correlate with clinical variables (Breitwieser et al., 2019; Steinegger and Salzberg, 2020; Gihawi et al., 2023; Ge et al., 2025; Salzberg et al., 2026). These problems are quantitatively less consequential for high-biomass fecal samples but can become decisive for low-biomass intratumoral and blood compartments, the same compartments that produced some of the most expansive recent claims.

### 9.2 The retraction setbacks

The landmark pan-cancer blood-and-tissue microbiome diagnostic study was retracted in 2024 after reanalysis argued that its discriminative signal was largely attributable to host-read misclassification and batch artifacts rather than cancer-specific microbial communities (Poore et al., 2020; Gihawi et al., 2023; Poore et al., 2024). A downstream prognosis and drug-response paper building on the same framework was subsequently retracted in 2025 (Hermida et al., 2022; Hermida et al., 2025). Most consequentially for CRC-relevant claims, a 2025 reanalysis of The Cancer Genome Atlas microbial data reported that, after stringent contamination and host-read controls, most previously claimed cancer-type-specific microbial signal did not survive (Ge et al., 2025). The arc is therefore a deflation of low-biomass pan-cancer signatures under contamination-aware scrutiny.

### 9.3 The defense and the unresolved state of the debate

The authors of the original report have not conceded the broader claim. In a 2024 response, they argued that cancer-associated microbial signals are recoverable across multiple independent methods and that some critiques conflate imperfect normalization with absence of biology (Sepich-Poore et al., 2024). The honest characterization is that the matter remains unresolved at the level of low-biomass signatures: critics have shown that specific high-profile signals are fragile to contamination control, while proponents maintain that a genuine, if smaller, signal persists. What it does take from the dispute is a methodological standard: any claim resting on low-biomass tissue or blood data must be evaluated against contamination-aware controls before it is treated as established (Salter et al., 2014; Poore et al., 2020; Hermida et al., 2025; Wang et al., 2025; Salzberg et al., 2026).

### 9.4 The absence of endorsed standards

Underlying the dispute there is a governance gap: no internationally endorsed standard operating procedures currently exist for intratumoral microbiome work across specimen collection, storage, nucleic-acid extraction, library preparation, sequencing, and data analysis. Recent reviews of the field note that inter-laboratory variation in extraction, library preparation, sequencing, and bioinformatic thresholding can materially alter whether a taxon is detected or interpreted as enriched (Cumbo and Niccolai, 2025; Wang et al., 2025). The absence of harmonized, ring-tested protocols restricts meta-analysis, hinders biomarker discovery, and sustains legitimate skepticism regarding the robustness of many published microbial signatures. Until consensus guidelines are established and adopted, reproducibility cannot be assumed for the low-biomass layer (Cumbo and Niccolai, 2025; Wang et al., 2025).

### 9.5 The robust-versus-fragile line for CRC

Robust evidence includes signals supported by biomass-adequate measurement and/or functional validation: fecal and luminal bacterial community signatures; mechanistically validated host-microbe interactions demonstrated in experimental systems, including colibactin genotoxicity, ETBF/BFT signaling, the *F. nucleatum* butyrate-HDAC-TBX21 axis, and the molecularly resolved *Candida-Fusobacterium* interaction; fecal mycobiome signatures and the Dectin-1/CARD9 immune axis; and metabolite-level read-outs that integrate community function (Chung et al., 2018; Coker et al., 2019; Dziubańska-Kusibab et al., 2020; Tang et al., 2023; Wang X. et al., 2024; Li J. et al., 2026). Fragile evidence includes intratumoral and blood-derived microbial signatures, low-biomass intratumoral fungal and viral signals, and any biomarker or mechanistic claim resting primarily on tissue or blood sequencing without negative controls, host-read filtering, biomass-aware thresholds, and orthogonal validation (Malik et al., 2018; Modoux et al., 2022; Narunsky-Haziza et al., 2022; Wan et al., 2022; Tang et al., 2023; Hermida et al., 2025). **Table 3** summarizes robust and fragile evidence.

**Table 3:** Robust versus fragile evidence in the CRC microbiome, a sorting framework for the reproducibility era.

Finding/claim	Evidence type	Basis for confidence
ROBUST — high-biomass and/or experimentally validated		
Fecal bacterial community signatures discriminate CRC across cohorts (Wirbel et al., 2019)	High-biomass fecal metagenomics; multi-cohort replication	Abundant microbial reads; replicated across independent international cohorts; contamination problem comparatively manageable
Multi-kingdom (bacterial-fungal) panels outperform single-kingdom for diagnosis (Liu et al., 2022)	Cross-cohort multi-kingdom metagenomics	Replicated multiple independent cohorts; effect consistent across populations
Colibactin causes CRC-associated mutational signatures (Pleguezuelos-Manzano et al., 2020; Severino and Ianiro, 2025)	Organoid causality + human genome signatures	Demonstrated in human intestinal organoids; signature reproduced *in vivo* and in patient genomes
*F. nucleatum* context-dependent therapeutic effects (Wang N. et al., 2024; Wang X. et al., 2024)	Experimentally validated mechanism (*in vitro*/*in vivo*)	Defined molecular mechanisms (butyrate/HDAC/TBX21; succinic acid; autophagy) shown in functional models, not inferred from sequencing
Interkingdom networks track immunotherapy response (Huang et al., 2023)	Multi-cohort network analysis	Network present in responders, absent in non-responders across cohorts; pattern more robust than single-taxon abundance
FRAGILE — low-biomass and/or contamination-vulnerable		
Pan-cancer blood/tissue microbial diagnostic signatures (original claim) (Poore et al., 2024)	Low-biomass tissue/blood sequencing	RETRACTED - invalidated by contamination and human-read misclassification; reanalysis found far smaller/absent signal
Prognosis/drug-response prediction from tumor microbiome (downstream) (Hermida et al., 2025)	Derived from low-biomass dataset above	RETRACTED 2025 - original results not reproducible with updated pipeline
CONTESTED — active, unresolved disagreement		
Whether anatomically internal tumors harbor a functionally relevant resident microbiome (Sepich-Poore et al., 2024; Ge et al., 2025)	Competing reanalyzes/defenses	Open disagreement among capable groups; gut (environment-continuous) tumors more plausible than sequestered organs

## 10 Translational opportunities and open questions

### 10.1 FMT as a multi-kingdom intervention

FMT is, by construction, a multi-kingdom intervention; it transfers bacteria, fungi, viruses, and metabolites together and is therefore one of the most direct ways to shift the ecosystem toward a treatment-sensitive state. The strategic interest in CRC is concentrated on MSS disease, where FMT is being tested as a means of converting non-responders to ICI responders. Several controlled efforts are active or have reported, including MSS CRC FMT-plus-immunotherapy studies such as RENMIN-215, a peer-reviewed open-label, single-arm phase II study, that FMT was combined with tislelizumab and fruquintinib in patients with refractory MSS metastatic CRC. Because the study lacked a control group and evaluated a three-component intervention, it does not isolate the contribution of FMT. A separate MD Anderson phase II study is evaluating FMT with re-introduction of pembrolizumab or nivolumab in patients with dMMR/MSI-H metastatic CRC who previously progressed on anti-PD-1 therapy (NCT04729322) and a recent systematic review aggregating FMT evidence across cancers (Zhao et al., 2023; Wekking et al., 2025; M.D. Anderson Cancer Center, 2026).

### 10.2 Phage therapy, FVT, and antifungal precision

Because phages set bacterial abundance and gene content, phage-based editing is the natural precision counterpart to the broad ecosystem reset of FMT. The *B. fragilis*-eliminating lytic phage that restores chemosensitivity remains a strong CRC proof-of-concept (Ding X. et al., 2025). Engineered approaches, including phage-FVT combinations, engineered phages, and bacterial chassis delivering phage-encoded payloads, are also advancing (Kenneth et al., 2025; Li HR. et al., 2025). On the fungal side, the predictive and mechanistic weight of the mycobiome supports interest in antifungal precision strategies and defined fungal-function approaches, although these remain preclinical (Ling et al., 2023; Rebeck et al., 2025; Ting et al., 2025). The take away message here is that any single-target intervention must be evaluated for network consequences, since editing one kingdom can restructure the others (Thingstad, 2000; Marantos et al., 2022; Brunner et al., 2024).

### 10.3 Multi-kingdom and ecological-state biomarkers

**Figure 2:**
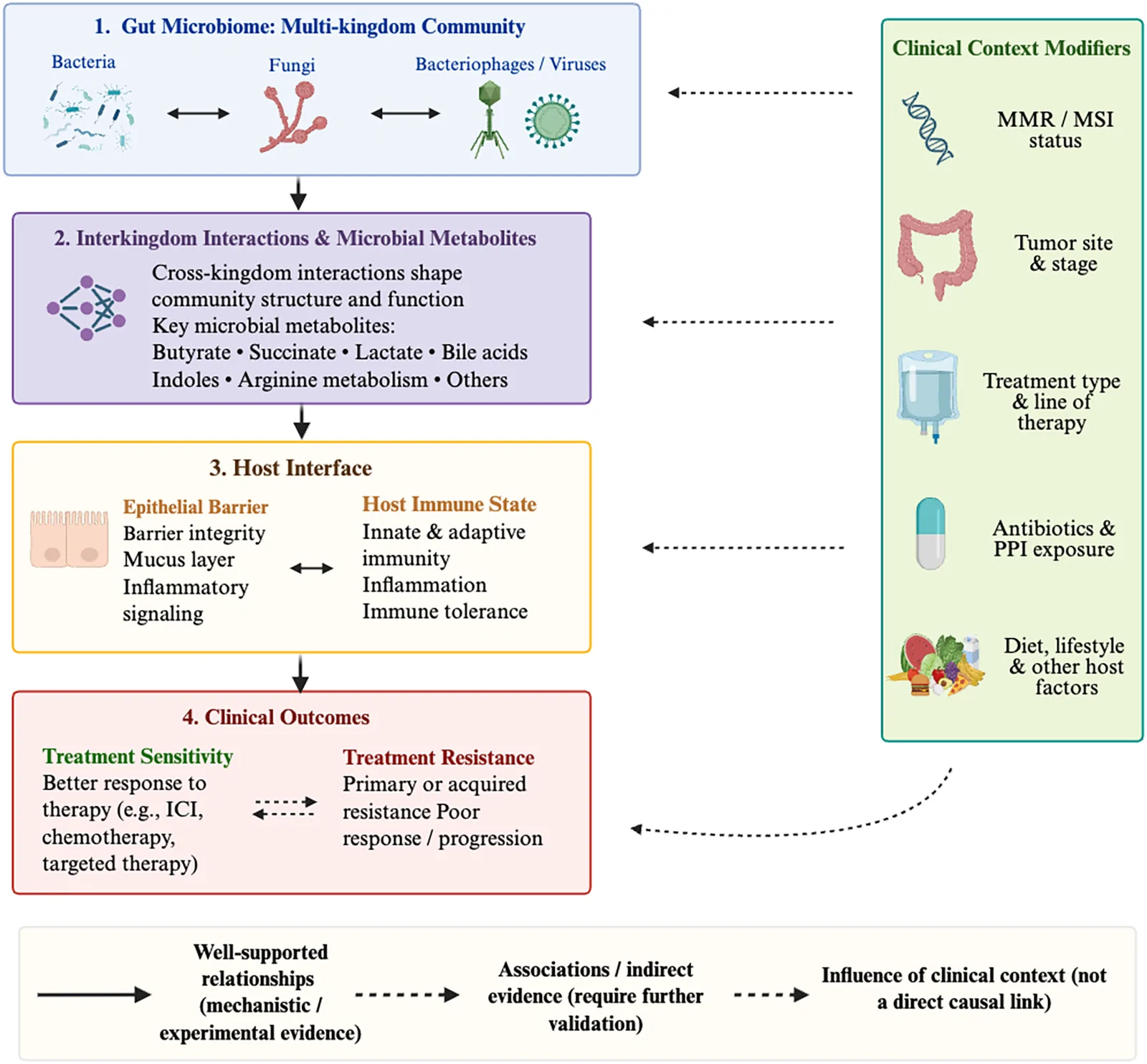
Simplified clinical framework linking the multi-kingdom microbiome to treatment response in colorectal cancer. Bacterial, fungal, and bacteriophage/viral communities interact directly and through microbial metabolites, with potential effects on epithelial barrier integrity and host immune state that may influence treatment sensitivity or resistance. These relationships are further shaped by clinical and host factors, including MMR/MSI status, tumor site and stage, treatment exposure, antibiotic use, and diet. The schematic is intended as a simplified translational framework; not all depicted relationships have been prospectively validated in patients with CRC.

The biomarker program implied by this review is a shift from taxon presence or absence toward multi-kingdom and ecological-state descriptors, including interkingdom-network connectivity, metabolite-output profiles, and diversity-and-resilience metrics. Predictive-modelling studies support this direction by showing that multi-kingdom features can outperform single-kingdom models (Huang et al., 2023; Qiao and Zhu, 2025). The underexplored archaeal compartment may add orthogonal markers: a multi-cohort analysis of more than 2, 000 fecal metagenomes found stage-associated shifts in methanogenic archaea and showed that a combined archaeal-bacterial model outperformed single-kingdom models for CRC discrimination, with CRC-depleted methanogens co-occurring with butyrate-producing bacteria (Li T. et al., 2025). To be clinically credible, any biomarker built on tissue or blood must be developed with contamination-aware controls; fecal and metabolite-based ecological markers are the more robust near-term path (Goodwin et al., 2011; Modoux et al., 2022; Wan et al., 2022; Hermida et al., 2025). Also, prospective biomarker development should be stratified by MMR/MSI status, tumor site, stage, treatment context, sample compartment, antibiotic exposure, and other major host variables. These interkingdom, host, and clinical relationships and their potential links to treatment response are summarized in a simplified translational framework in **Figure 2**.

A recent review centered on *Fusobacterium nucleatum* illustrates this translational continuum, from its potential incorporation into multimodal biomarker panels for CRC detection and patient stratification to experimental targeting through antibiotics, bacteriophages, nanodrugs, dietary modulation, and microbiota-derived products; however, the review also emphasizes that most therapeutic evidence remains preclinical and that, at the time of publication, no interventional clinical study had directly evaluated *F. nucleatum*-targeted treatment in CRC (Incognito et al., 2025).

From a clinical perspective, microbiome-derived biomarkers remain at different and generally early stages of development. Replicated fecal and multi-kingdom signatures support further investigation for CRC detection, whereas microbiome-based predictors of treatment response are derived predominantly from retrospective, pan-cancer, or computational analyses (Huang et al., 2023; Huang et al., 2023; Chen X. et al., 2025; Li T. et al., 2025; Qiao and Zhu, 2025). Interventional evidence is similarly limited to small, early-phase, and frequently multi-component studies, such as RENMIN-215, in which the independent contribution of FMT cannot be determined (Zhao et al., 2023). Accordingly, no microbiome-derived assay currently has sufficient evidence to guide routine treatment selection in CRC. Clinical implementation will require a predefined intended use, a locked and reproducible assay, external multicenter validation, and demonstration of incremental clinical value beyond established variables such as MMR/MSI status, tumor stage and location, treatment line, and standard molecular biomarkers. Development and validation cohorts should additionally account for antibiotic and proton-pump inhibitor exposure, diet, geography, sample compartment, and other major host factors.

### 10.4 Open questions

Four research gaps remain open. First, no completed prospective CRC trial has yet established whether multi-kingdom microbiome profiling can predict or modify ICI response in CRC; current predictive models remain largely retrospective, and prospective validation is the field’s most important missing experiment. Prospective longitudinal studies are needed to determine how ecological states evolve throughout the adenoma and carcinoma sequence and during therapy, rather than relying primarily on cross-sectional observations. Also, mechanistic causality should increasingly be tested using synthetic microbial communities, organoids, gnotobiotic animal models, and spatially resolved experimental systems capable of reconstructing defined interkingdom interaction networks (Huang et al., 2023; Qiao and Zhu, 2025). Second, causality for most cross-kingdom interactions still needs to be tested in gnotobiotic, organoid, and spatially resolved systems rather than inferred from association alone (Chen X. et al., 2025; Li J. et al., 2026). Third, early-onset CRC mechanisms, including developmental and early-life microbial exposures that may seed later mutational and ecological trajectories, remain incompletely mapped (Luevano et al., 2025; Severino and Ianiro, 2025). Fourth, the standardization gap discussed above remains a precondition for trusting the low-biomass layer (Gihawi et al., 2023; Cumbo and Niccolai, 2025; Ge et al., 2025; Wang et al., 2025). Progress will depend as much on methodological rigor as on new biology.

Several barriers must be addressed before microbiome-based strategies can enter routine CRC care. Pre-analytical procedures require harmonization across sample type, collection timing, storage, and nucleic-acid extraction, while analytical reproducibility remains sensitive to sequencing platforms, reference databases, host-read filtering, contamination control, normalization, and bioinformatic pipelines (Salter et al., 2014; Davis et al., 2018; Cumbo and Niccolai, 2025; Wang et al., 2025). Future biomarker studies should therefore use prespecified analytical workflows, appropriate controls, and independent external validation. Most importantly, clinical validity and utility must be demonstrated prospectively in multicenter cohorts representing the intended-use population, using predefined endpoints and comparison with established clinical and molecular predictors. Microbiome testing will require evidence of incremental clinical value rather than discrimination or biological plausibility alone (Porcari et al., 2025).

## 11 Discussion

### 11.1 Summary of finding

This review advances two central conclusions. First, the multi-kingdom CRC microbiome is clinically and mechanistically relevant, but its interpretation requires a clear distinction between robust biological evidence and fragile low-biomass signal. Fecal community signatures, experimentally validated host-microbe mechanisms, metabolite-level read-outs, and functional fungal immunology currently stand on firmer ground than intratumoral or blood-derived microbial signatures that remain vulnerable to contamination, host-read misclassification, and batch effects. Second, within this more disciplined evidence base, interkingdom interaction rather than individual taxonomic presence often provides the more informative framework for understanding therapeutic response and resistance.

This interaction-centered view helps explain why the same organism can produce divergent clinical associations across contexts. The *F. nucleatum* paradox, in which one organism may contribute to immune suppression, chemoresistance, ICI resistance, or anti-PD-1 sensitization depending on metabolite output and immune context, is difficult to reconcile under a purely taxon-centric model but becomes more coherent under an ecological one. The practical implication is that CRC microbiome research should increasingly measure ecological state, predict from interkingdom networks, and intervene at the level of ecosystem function rather than targeting single organisms alone.

Translation will require prospective validation, particularly in MSS CRC, where the clinical need is greatest. FMT, phage-based editing, metabolite modulation, and antifungal precision strategies are promising, but they should be evaluated as ecosystem-level interventions with attention to network consequences across kingdoms. At the same time, contamination-aware pipelines and standardized low-biomass protocols should become default requirements for tissue- and blood-based microbiome studies. A multi-kingdom microbiome framework, applied with methodological discipline, may provide a more reliable and clinically useful approach to CRC microbiome biology than taxonomic cataloguing alone.

### 11.2 Relationship to prior multi-kingdom syntheses

The multi-kingdom organization of the CRC microbiome has been recognized in prior work, and the present synthesis is not the first to argue that bacteria, fungi, and viruses should be considered together. Earlier multi-kingdom analyses established that fungal and viral signatures add discriminatory value beyond the bacteriome, and recent syntheses have framed CRC as a biofilm-structured, microbiome-driven ecosystem disease in which polymicrobial consortia, rather than isolated taxa, constitute the tumor-relevant unit. Our contribution is therefore positioned not as a claim of priority over these efforts but as a difference in analytic emphasis. Where consortium-centered frameworks foreground the physical and structural organization of polymicrobial communities, the framework developed here treats the interkingdom interaction itself as the functional and predictive unit, and it pairs that claim with an explicit evidentiary sorting principle that separates robust fecal and mechanistic signals from fragile low-biomass intratumoral observations. The second distinguishing proposal is that therapeutic resistance may sometimes be interpreted as a treatment-selected ecological state. This proposal extends existing consortium frameworks but remains conceptual until longitudinal CRC studies directly demonstrate coordinated state transitions and their functional consequences. These two claims are intended to refine, rather than displace, the consortium and ecosystem models that precede this review.

### 11.3 Limitations

Several limitations qualify the conclusions of this review. First, the evidence base is markedly uneven across kingdoms: the bacteriome rests on replicated cross-cohort data, the mycobiome on a mixture of robust mechanistic and more fragile taxonomic findings, and the virome on largely correlative and hypothesis-generating observations, with no CRC virome-immunotherapy-response data currently available. Comparisons across kingdoms profiled with assays of unequal reliability should therefore be interpreted with caution. Second, much of the interaction-level evidence that motivates the central thesis derives from preclinical and ex vivo systems, and the molecularly resolved cross-kingdom mechanisms highlighted here require prospective confirmation in human CRC cohorts before they can guide clinical decision-making. Third, the interaction-centered and ecological-state framework remains, at present, a conceptual model: the field still lacks endorsed minimum methodological standards for low-biomass and multi-kingdom studies, validated ecological-state biomarkers, and adequately powered interventional trials. Finally, as a narrative rather than systematic review, this synthesis reflects a selective rather than exhaustive appraisal of a rapidly evolving literature and is subject to the attendant selection bias. These limitations underscore that the framework proposed here is intended to structure future investigation rather than to support immediate changes in clinical practice.

### 11.4 Conclusions

In summary, the CRC microbiome is most informatively viewed not as a catalog of individual taxa but as an interacting multi-kingdom ecosystem whose clinically relevant outputs, including therapeutic response and resistance, emerge from interkingdom interactions shaped by host physiology. Read through a contamination-aware lens that distinguishes robust from fragile evidence, this interaction-centered and ecological-state framework offers a more coherent account of phenomena such as the *F. nucleatum* paradox and a more rational basis for biomarker development and ecosystem-level intervention. Realizing this potential will require prospective validation in well-controlled human cohorts, standardized low-biomass methodology, and interventional trials designed to test ecological rather than single-organism hypotheses.

Beyond microbial composition alone, the host physiological landscape ultimately determines which interkingdom interactions can emerge and persist. Oxygen gradients, epithelial barrier integrity, nutrient availability, immune-cell organization, and local metabolic conditions act as master ecological regulators that shape microbial cooperation, competition, and signaling. Accordingly, host physiology should be viewed not merely as the background against which microbial interactions occur, but as the principal determinant of their biological and clinical consequences.

We propose that the next generation of colorectal cancer microbiome research should move beyond cataloguing individual microorganisms toward defining ecological interaction modules that directly regulate host physiology, immune function, and therapeutic response. Such a framework integrates microbial composition with spatial organization, metabolic exchange, and host physiology while maintaining rigorous contamination-aware methodology. By focusing on ecosystem function rather than taxonomy alone, future studies may generate more reproducible biomarkers and more effective microbiome-directed therapeutic strategies for colorectal cancer.

## Glossary

AhR: aryl hydrocarbon receptor
AMG: auxiliary metabolic gene
AUC: area under the curve
BCL10: B-cell lymphoma/leukemia 10
BFT: Bacteroides fragilis toxin
BIRC3: baculoviral IAP repeat-containing protein 3
CARD9: caspase recruitment domain-containing protein 9
CCL5: C-C motif chemokine ligand 5
CLEC7A: C-type lectin domain-containing 7A
CRC: colorectal cancer
CXCL10: C-X-C motif chemokine ligand 10
dMMR: deficient mismatch repair
EMT: epithelial–mesenchymal transition
ETBF: enterotoxigenic Bacteroides fragilis
FadA: Fusobacterium adhesin A
Fap2: Fusobacterium autotransporter protein 2
FMT: fecal microbiota transplantation
Fna: Fusobacterium nucleatum subsp. animalis
FVT: fecal virome transplantation
FXR: farnesoid X receptor
GSDME: gasdermin E
HDAC: histone deacetylase
HGT: horizontal gene transfer
HIF-1α: hypoxia-inducible factor 1 alpha
ICI: immune checkpoint inhibitor
ID-pks: insertion–deletion mutational signature associated with the pks genotoxic pathway
IFN-β: interferon beta
IL: interleukin
ITS: internal transcribed spacer
MALT1: mucosa-associated lymphoid tissue lymphoma translocation protein 1
MAMPs: microbial-associated molecular patterns
MDSC: myeloid-derived suppressor cell
MMR: mismatch repair
MSI: microsatellite instability
MSI-H: microsatellite instability-high
MSS: microsatellite stable
NF-κB: nuclear factor kappa B
NLR: NOD-like receptor
NLRP3: NOD-, LRR- and pyrin domain-containing protein 3
PD-1: programmed cell death protein 1
PD-L1: programmed death-ligand 1
pMMR: proficient mismatch repair
pks: polyketide synthase
RALB: RAS-like proto-oncogene B
rRNA: ribosomal RNA
SBS-pks: single-base substitution mutational signature associated with the pks genotoxic pathway
SCFA: short-chain fatty acid
STAT3: signal transducer and activator of transcription 3
SYK: spleen tyrosine kinase
TBX21: T-box transcription factor 21
TGR5: Takeda G protein-coupled receptor 5
TIGIT: T-cell immunoreceptor with immunoglobulin and ITIM domains
TLR: Toll-like receptor
TNF-α: tumor necrosis factor alpha
VLP: virus-like particle
YAP: Yes-associated protein
